# Supplementary material for: Analysis of novel therapeutic targets and construction of a prognostic model for hepatocellular carcinoma
Source: PeerJ. 2025 Aug 22;13:e19899. doi: 10.7717/peerj.19899 (PMC12377355; doi:10.7717/peerj.19899)
Supplement: Supplemental Information 1 [file peerj-13-19899-s001.docx]

Western Blot results:

| Name | Molecular weight | Original bands/gels  M THLE-2 MHCC97H |
| --- | --- | --- |
| CBS | 72kda  100 kDa  70 kDa  55 kDa | 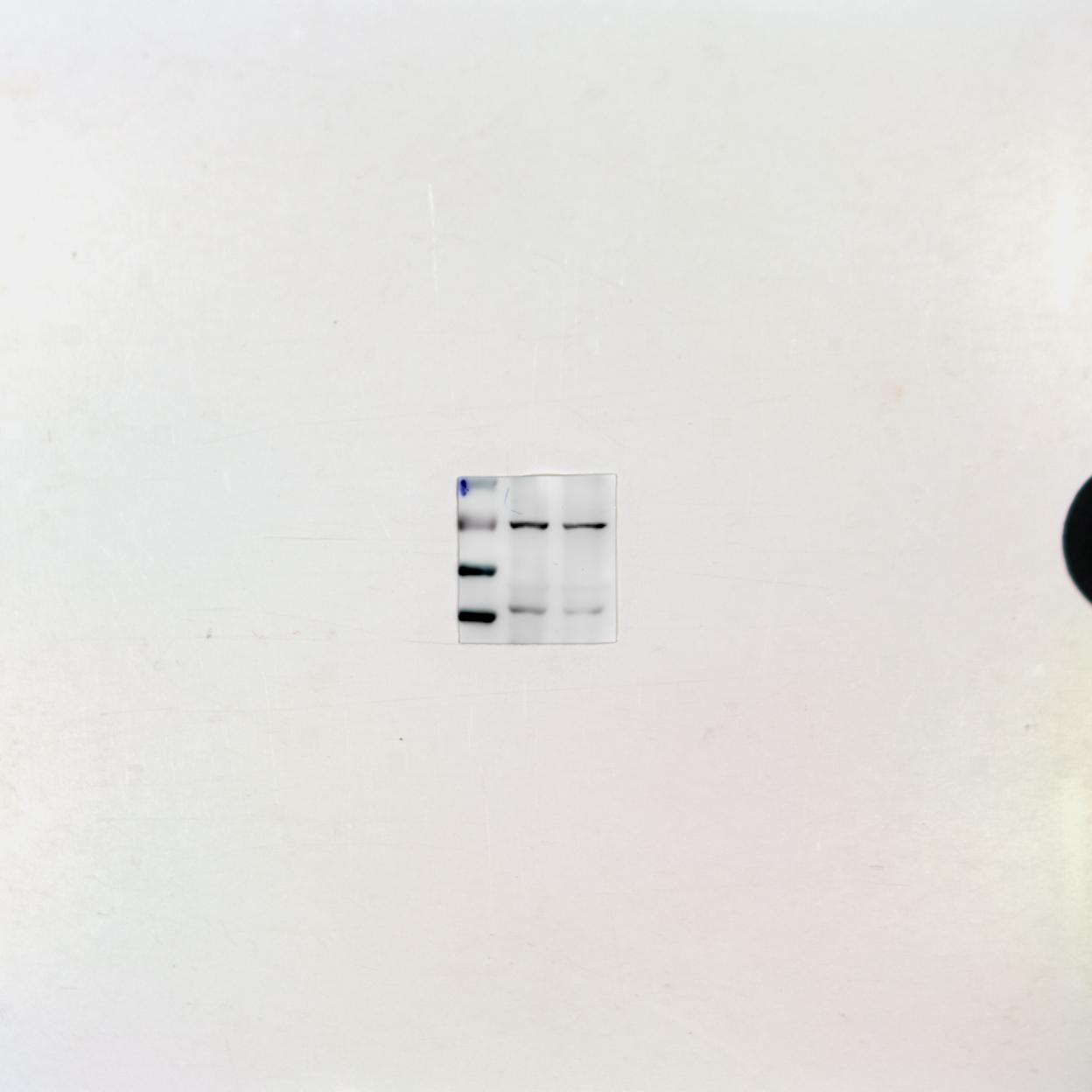  40 kDa |
| β-actin | 42kDa |  |
